# A clinical prediction nomogram to assess risk of colorectal cancer among patients with type 2 diabetes

**DOI:** 10.1038/s41598-020-71456-2

**Published:** 2020-09-01

**Authors:** Lu-Huai Feng, Tingting Su, Kun-Peng Bu, Shuang Ren, Zhenhua Yang, Cheng-En Deng, Bi-Xun Li, Wei-Yuan Wei

**Affiliations:** 1 Department of Comprehensive Internal Medicine, The Affiliated Tumor Hospital of Guangxi Medical University, Nanning, China; 2 Department of Urology, The Affiliated Tumor Hospital of Guangxi Medical University, Nanning, China; 3 Department of Nephrology, The First Affiliated Hospital of Guangxi Medical University, Nanning, China; 4 Department of Gastrointestinal Surgery, The Affiliated Tumor Hospital of Guangxi Medical University, Nanning, China; 5 Department of ECG Diagnostics, The People’s Hospital of Guangxi Zhuang Autonomous Region, Nanning, China

**Keywords:** Cancer, Diseases, Gastroenterology, Health care, Oncology

## Abstract

Colorectal cancer remains a major health burden worldwide and is closely related to type 2 diabetes. This study aimed to develop and validate a colorectal cancer risk prediction model to identify high-risk individuals with type 2 diabetes. Records of 930 patients with type 2 diabetes were reviewed and data were collected from 1 November 2013 to 31 December 2019. Clinical and demographic parameters were analyzed using univariable and multivariable logistic regression analysis. The nomogram to assess the risk of colorectal cancer was constructed and validated by bootstrap resampling. Predictors in the prediction nomogram included age, sex, other blood-glucose-lowering drugs and thiazolidinediones. The nomogram demonstrated moderate discrimination in estimating the risk of colorectal cancer, with Hosmer–Lemeshow test *P* = 0.837, an unadjusted C-index of 0.713 (95% CI 0.670–0.757) and a bootstrap-corrected C index of 0.708. In addition, the decision curve analysis demonstrated that the nomogram would be clinically useful. We have developed a nomogram that can predict the risk of colorectal cancer in patients with type 2 diabetes. The nomogram showed favorable calibration and discrimination values, which may help clinicians in making recommendations about colorectal cancer screening for patients with type 2 diabetes.

## Introduction

Colorectal cancer is one of the most common and aggressive clinical gastrointestinal cancer that causes a serious threat to human life and health^1^, accounting for approximately seven hundred thousand annual deaths worldwide^2^. Despite the rapid development of diagnostic and treatment methods, the 5-year survival rate for colorectal cancer is ≈ 50% overall^3^, although this rate for colorectal cancer diagnosed in the early stages is > 90%^4^. The reason for this abysmal prognosis is that the vast majority of colorectal cancer patients are diagnosed at an advanced stage^5^. Therefore, early diagnosis of colorectal cancer is particularly important. Unfortunately, huge resources have been invested in the prevention and early diagnosis of colorectal cancer, but there is a limitation on the effective and early diagnosis of colorectal cancer^6^.

Screening can reduce the incidence and mortality of colorectal cancer by 30% and 50%, respectively^7^. Current screening methods for colorectal cancer mainly include flexible sigmoidoscopy, colonoscopy, fecal occult blood testing, double-contrast barium enema, stool DNA testing, and computed tomographic colonography^8^. However, these examinations are invasive or time-consuming or expensive, and it is not feasible to screen the general population for colorectal cancer. To improve the early diagnosis of this disease, we need to broaden our understanding of it.

International screening guidelines recommend that screening for colorectal cancer starts at 50 years of age for the average risk group and 50–75 years of age as the target age group for colorectal cancer screening^9,10^. It is worth mentioning that diabetes is one of the indicators of the average risk group evaluation in China's guidelines. Many epidemiological studies have reported that type 2 diabetic patients are at a higher risk of developing colorectal cancer compared with non-diabetic patients^11–13^. A meta-analysis showed that the risk of developing colorectal cancer in type 2 diabetic patients is 20–40% greater than non-diabetic individuals^14^. Diabetes may influence the neoplastic process by several mechanisms, including hyperinsulinemia (either endogenous due to insulin resistance or exogenous due to administered insulin or insulin secretagogues), hyperglycemia, or chronic inflammation^13^. However, due to the high incidence of type 2 diabetes mellitus in the general population and the lack of a low-risk and low-cost colorectal cancer screening method, it is not feasible to conduct mass colorectal cancer screening in all patients with type 2 diabetes mellitus. Besides, targeted screening for colorectal cancer (e.g., using flexible sigmoidoscopy) may be feasible if diabetic individuals at the highest risk for colorectal cancer could be identified.

Based on existing knowledge, a variety of epidemiological and/or clinical characteristics (e.g., weight changes^15^, lifestyle factors such as smoking^16^, medications such as metformin, insulin^17^) can be used to predict the risk of colorectal cancer in patients with type 2 diabetes mellitus. Similar risk prediction models exist for other diseases, such as diabetic nephropathy^18^ and pancreatic ductal adenocarcinoma-associated diabetes mellitus^19^. To date, epidemiological studies of colorectal cancer predictors associated with type 2 diabetes mellitus have been limited to the assessment of individual predictors. The nomogram has been accepted as a reliable tool to create a simple intuitive graph for a statistical predictive model that quantifies the risk of a clinical event^20,21^. This study aimed to identify a combination of variables that would enable a highly accurate prediction of colorectal cancer in patients with type 2 diabetes. In addition, a nomogram for predicting the risk of colorectal cancer was constructed for patients with type 2 diabetes to effectively identify patients at high risk for colorectal cancer.

## Material and methods

This retrospective study was performed with approval from the Ethics Committee of the Affiliated Tumor Hospital of Guangxi Medical University (Approval N. LW2020010). The data are anonymous, and the requirement for informed consent was therefore waived. This study was conducted per the Helsinki Declaration.

### Patients

This was a retrospective study. Electronic records were used to identify patients diagnosed colorectal cancer with type 2 diabetes between November 1, 2013, and September 31, 2019, at the Affiliated Tumor Hospital of Guangxi Medical University. Patients with a clear diagnosis of type 2 diabetes before the diagnosis of colorectal cancer were included in the study, while those with two or more types of cancers were excluded.

### Data collection

Participants were divided into colorectal cancer group (n = 118) and non-colorectal cancer group (n = 812). Clinical parameters and demographic data were collected at the time of colorectal cancer diagnosis, including age, sex, duration of diabetes (months), use of blood glucose-lowering drugs, history of smoking, body mass index (BMI), and family history of colorectal cancer. The duration of diabetes was defined as the period from first diagnosis of type 2 diabetes to the diagnosis of malignancy. The diagnosis of type 2 diabetes in our institution complied with World Health Organization criteria^22^. Blood glucose-lowering drugs included insulin, thiazolidinediones, alpha-glucosidase inhibitors, sulfonylureas and other blood-glucose-lowering drugs known as Chinese medicine or proprietary Chinese medicine of blood glucose-lowering drugs (Note: no patients in our study cohort used DPP-4 inhibitors, GLP-1 receptor agonists and SGLT2 inhibitors). Family history of colorectal cancer was defined as more than one first-degree relative with colorectal carcinoma. Smoking was defined as the number of packs of cigarettes (1 pack = 20 cigarettes) smoked daily per year, including current smokers and ex-smokers^23^.

### Statistical analysis

The statistical analyses were performed in SPSS version 26.0 (IBM Corp, Armonk, NY, USA), and graphics produced with R software (rms^24^ in R version 3.6.2; https://www.r-project.org/). For all analyses, statistical significance was set at *P* < 0.05. Continuous variables are presented as median and range (M (P25, P75)) or mean ± standard deviation. Categorical variables are presented as whole numbers and proportions. Differences between the groups were assessed using the chi-square test, *t*-test, or Wilcoxon rank-sum test. The assumption of linearity in the logit for the continuous variable was assessed and the significance of each variable in the cohort was assessed by univariate logistic regression to determine the independent predictor for colorectal cancer. All variables with *P* < 0.05 in the univariate logistic analyses were further assessed by multivariable logistic regression using backward stepwise selection. A clinical prediction nomogram to assess risk of colorectal cancer was constructed based on the results from the final multivariable logistic regression using the R software. Multicollinearity was checked before determining the final model.

Calibration curves were plotted to calibrate the nomogram. The C-index was used to evaluate discriminative ability, ranging from 0.5 (absence of discrimination) to 1 (perfect discrimination)^25^. In addition, the nomogram was subjected to 1000 bootstrap resamples for internal validation to assess predictive accuracy^26^.

Decision curve analysis was conducted by using the R software (rmda^27^ in R version 3.6.2) to determine the clinical utility of the nomogram by quantifying the net benefit at different threshold probabilities. The net benefit was calculated by subtracting the proportion of false positives from the proportion of true positives, and weighting by the relative harm of foregoing treatment compared with the negative consequences of an unnecessary treatment^28^.

## Results

A total of 930 patients were enrolled to develop and validate our predictive nomogram model. The clinical characteristics of patients are summarized in Table 1. Of the 930 patients, 544 (58.5%) were males and 386 (41.5%) were females, with the age of patients ranging from 33 to 86 years old (median: 60 years). The colorectal cancer group comprised of 118 (12.7%) patients. Overall, compared with the No-Colorectal cancer group, older patients, patients with longer duration of diabetes, male patients, and patients using thiazolidinediones are more likely to develop colorectal cancer.

**Table 1:** Clinical characteristics of patients.

Variable	Colorectal cancer group (n = 118)	No-Colorectal cancer group (n = 812)	*P *value
Age, years	63 (56,69)	59 (53,65)	< 0.001
Duration of diabetes, month	46 (12,84)	24 (0,68)	< 0.001
**Sex, N (%)**			
Male	98 (83.1)	446 (54.9)	< 0.001
Female	20 (16.9)	366 (45.1)	
BMI, kg/m^2^	23.0 (21.6, 25.1)	23.5 (21.5, 25.9)	0.139
Smoking, yes, N (%)	45 (38.1)	273 (33.6)	0.334
Family history of colorectal cancer, yes, N (%)	89 (13.6%)	34 (12.4%)	0.615
**Blood glucose-lowering drugs**			
Insulin, yes, N (%)	27 (22.9)	138 (17.0)	0.118
Thiazolidinediones, yes, N (%)	7 (5.9)	9 (1.1)	< 0.001
Alpha glucosidase inhibitors, yes, N (%)	18 (15.3)	90 (11.1)	0.186
Sulfonylureas, yes, N (%)	11 (9.3)	118 (14.5)	0.126
Metformin, yes, N (%)	28 (23.7)	138 (17.0)	0.074
Other blood-glucose-lowering drugs	23 (19.5)	100 (12.3)	0.032
Combination of oral drugs and insulin, yes, N (%)	6 (5.1)	28 (3.4)	0.376
Combination of oral drugs, yes, N (%)	20 (16.9)	88 (10.8)	0.053

Other blood-glucose-lowering drugs: defined as Chinese medicine or roprietary Chinese medicine of blood-glucose-lowering drugs.

No patients in our study cohort used DPP-4 inhibitors, GLP-1 receptor agonists, and SGLT2 inhibitors.

*BMI* body mass index.

### Selected predictors for model

After univariate logistic analysis (Table 2), variables including age, duration of diabetes, sex, BMI, thiazolidinediones and other blood-glucose-lowering drugs were included in the multivariable logistic regression analysis. The multivariable logistic analysis based on the backward stepwise approach demonstrated that the occurrence of colorectal cancer was significantly related to age (*P* < 0.001), sex (*P* < 0.001), other blood-glucose-lowering drugs (*P* = 0.024), and thiazolidinediones (*P* < 0.001) (Table 3). Overall, compared with the No-Colorectal cancer group, T2DM patients in the following categories (male, with longer duration of diabetes, older, using thiazolidinediones and other blood-glucose-lowering drugs) are at higher risk of colorectal cancer. In particular, there were no statistical differences in insulin and metformin in this study.

**Table 2:** Univariate logistic regression analysis of the predictor of Colorectal Cancer.

Variable	β	Odds ratio (95% CI)	*P* value
Age, years	0.043	1.044 (1.023–1.066)	< 0.001
Duration of diabetes, month	0.003	1.003 (1.000–1.005)	0.041
Family history of colorectal cancer	0.132	1.141 (0.722–1.805)	0.572
Sex	− 1.392	0.249 (0.151–0.410)	< 0.001
BMI, kg/m^2^	− 0.082	0.922 (0.914–0.929)	< 0.001
Smoking, yes	0.196	1.217(0.817–1.814)	0.335
**Blood-glucose-lowering drugs**			
Insulin, yes	0.371	1.449 (0.908–2.311)	0.119
Thiazolidinediones, yes,	1.728	5.627 (2.055–15.409)	0.001
Alpha glucosidase inhibitors, yes,	0.367	1.444 (0.835–2.497)	0.188
Sulfonylureas, yes	− 0.503	0.605 (0.315–1.159)	0.130
Metformin, yes,	0.418	1.519 (0.957–2.412)	0.076
Other blood-glucose-lowering drugs	0.545	1.724 (1.044–2.846)	0.033
Combination of oral drugs and insulin, yes	0.405	1.500 (0.608–3.703)	0.379
Combination oforal drugs, yes	0.518	1.679 (0.989–2.851)	0.055

Other blood-glucose-lowering drugs: defined as Chinese medicine or proprietary Chinese medicine of blood-glucose-lowering drugs.

No patients in our study cohort used DPP-4 inhibitors, GLP-1 receptor agonists and SGLT2 inhibitors.

*BMI* body mass index.

**Table 3:** Multivariate logistic multivariable regression analysis of the predictor of colorectal cancer.

Variable	β	Odds ratio (95% CI)	*P* value
Sex	− 1.416	0.243 (0.144–0.408)	< 0.001
Druation of diabetes	0.001	1.001 (0.998–1.004)	0.397
Age	0.044	1.045 (1.022–1.068)	< 0.001
BMI	− 0.028	0.972 (0.910–1.039)	0.403
Thiazolidinediones	2.131	8.426 (2.784–25.508)	< 0.001
Other blood-glucose-lowering drugs	0.605	1.831 (1.083–3.098)	0.024

Other blood-glucose-lowering drugs: defined as Chinese medicine or proprietary Chinese medicine of blood-glucose-lowering drugs.

*BMI* body mass index.

### Predictive nomogram for the risk of colorectal cancer

**Figure 1:**
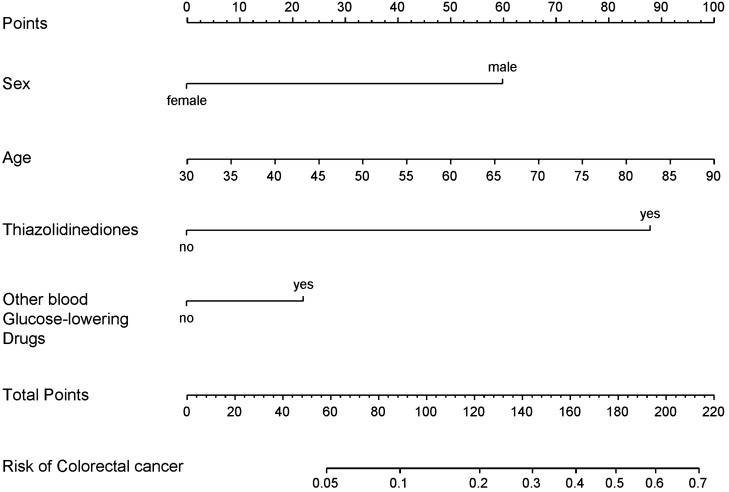
Nomogram developed with sex, age, other blood-glucose-lowering drugs, and thiazolidinediones incorporated.

Based on the final multivariate logistic regression, a nomogram was established that included 4 significant predictors for colorectal cancer prediction (Fig. 1). A total score was generated using age, sex, other blood-glucose-lowering drugs, and thiazolidinediones. Briefly, the nomogram finds the position of each variable on the corresponding axis, draws a line to the points axis for the number of points, adds the points from all of the variables, and draws a line from the total points axis to determine the colorectal cancer probabilities at the lower line of the nomogram.

### Performance of the nomogram

**Figure 2:**
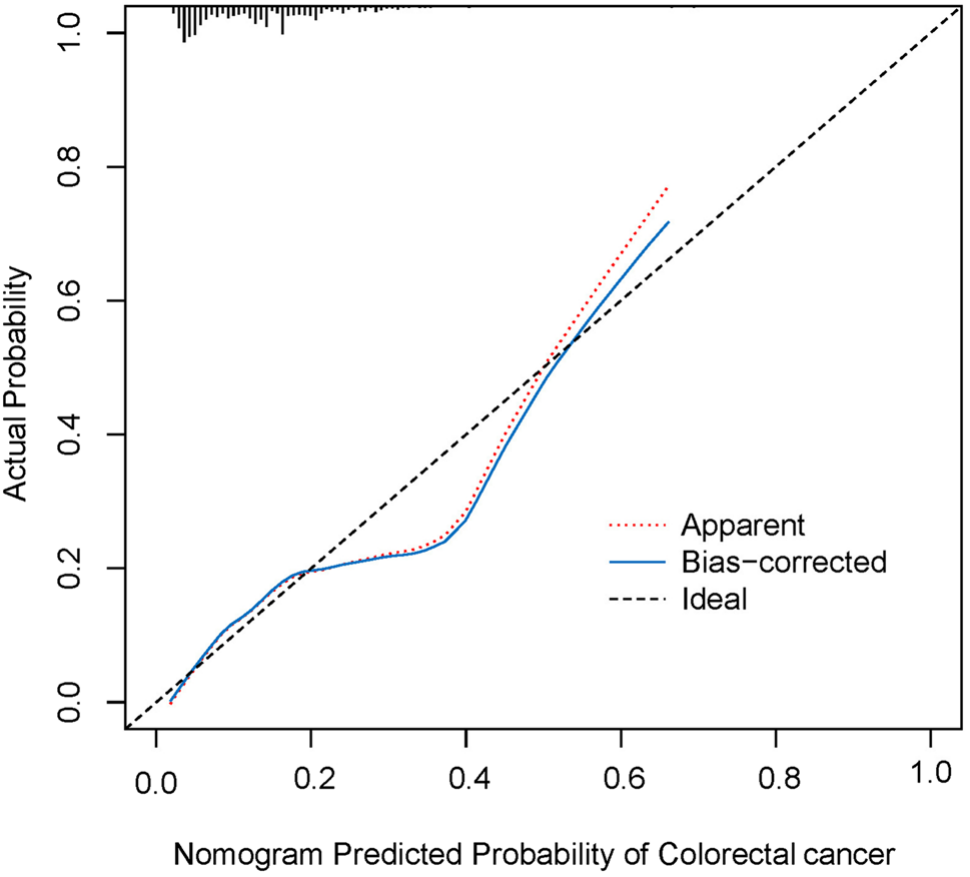
Calibration curves for the nomogram. The red dotted line represents the entire cohort (n = 930), while the blue solid line is the result after bias-correction by bootstrapping (1000 repetitions), indicating nomogram performance.

To validate the performance of the resulting nomogram, we performed internal validation by using the bootstrap method with 1000 repetitions. The nomogram demonstrated moderate discrimination in estimating the risk of colorectal cancer, with Hosmer–Lemeshow test P = 0.837, an unadjusted C index of 0.713 (95% CI 0.670–0.757), and a bootstrap-corrected C index of 0.708, indicating favorable discrimination. In addition, overall calibration plots were outstanding for the occurrence of colorectal cancer between the probabilities predicted by the nomogram and actual probabilities (Fig. 2).

### Clinical use of nomogram

**Figure 3:**
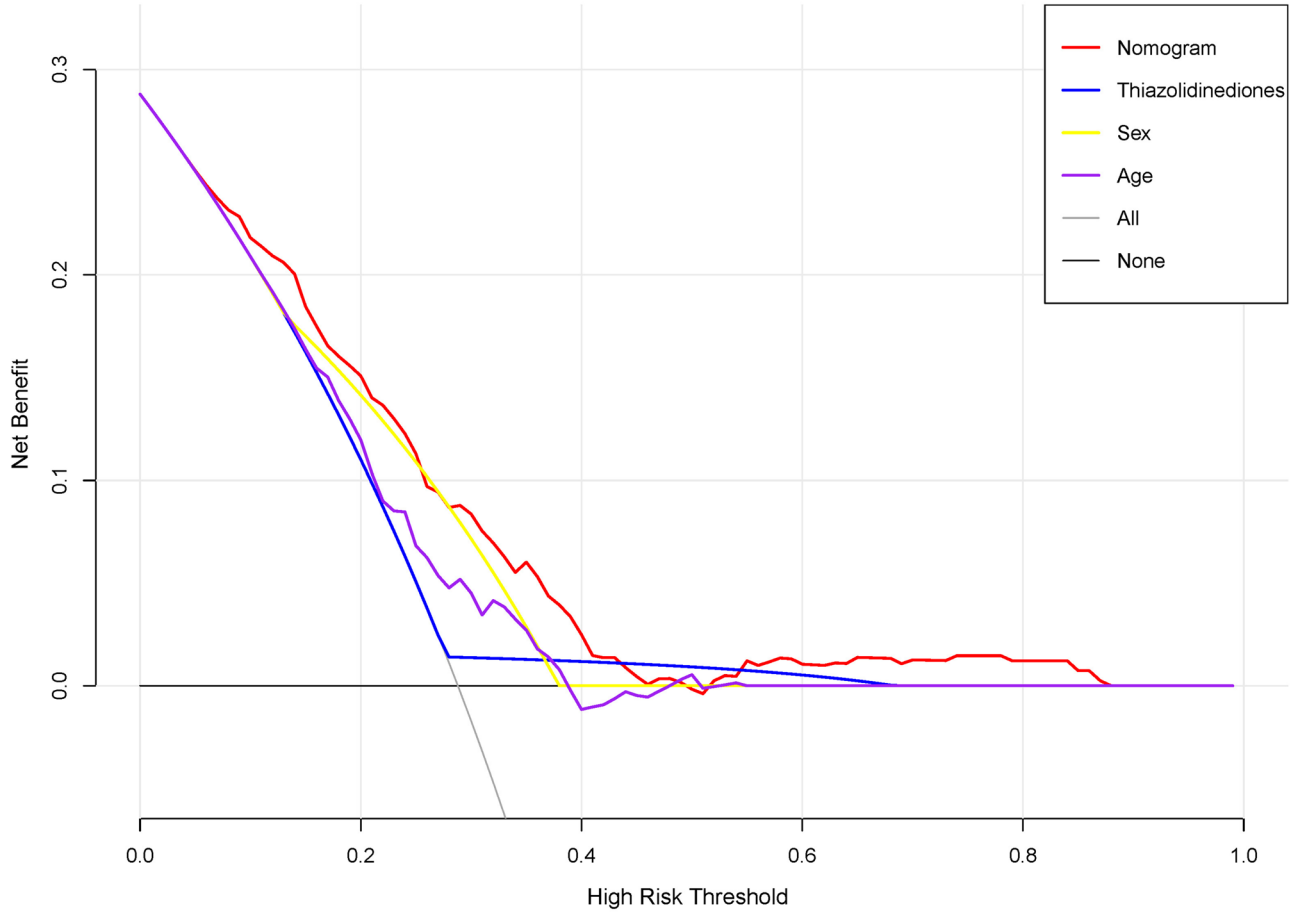
Decision curve analysis for the nomogram, thiazolidinediones, sex and age. The x-axis shows the threshold probability, and the y-axis measures the net benefit. The red line represents the nomogram. The blue line represents the model with thiazolidinediones. The yellow line represents the model with sex. The purple line represents the model with age.

The decision curve analysis (DCA) for the prediction nomogram and that for the model with single predictor is presented in Fig. 3. The final DCA showed that if the threshold probability of patients or clinicians is between 10 and 45%, screening strategies based on our nomogram’ colorectal cancer risk estimates resulted in superior net benefit than screen-none or screen-all strategies. Within this range, the predictive effect of nomogram is better than that of a single predictor, respectively.

## Discussion

We developed and internally validated a simple intuitive statistical predictive model to quantify colorectal cancer-associated diabetes mellitus among patients with type 2 diabetes mellitus. The model was developed in a diabetes type 2 cohort and focused on demographic and clinical variables that would be routinely available at the time of diabetes diagnosis. In estimating the risk of colorectal cancer, the final model showed good discrimination and calibration; with the C-index was 0.713 (0.734 via bootstrapping validation) and the Hosmer–Lemeshow test showed no significant statistic (*P* = 0.837).

In recent years, colorectal cancer has increased in incidence and a tendency to start at a younger age^29^, accounting for nearly seven hundred thousand deaths worldwide annually^2^. Radical surgical excision of early-stage colorectal cancer results in a high 5-year survival rate of up to 90%, but in metastatic colorectal cancer, this rate decreases to 15%^4^. Early diagnosis and treatment play an important role in reducing mortality and morbidity in colorectal cancers. Although colorectal cancer morbidity and mortality are high, universal screening using biomarkers, such as a carcinoembryonic antigen or clinical tests (eg., flexible sigmoidoscopy, colonoscopy, fecal occult blood testing or computed tomographic colonography) is not feasible. While highly specific and sensitive, the modalities of clinical diagnosis entail substantial risk and/or cost. Therefore, novel prediction models and screening methods for detecting asymptomatic early-stage disease are urgently needed, which would be both efficient and cost-effective^30^.

Epidemiological studies suggest DM is associated with an increased risk of colorectal cancer, especially T2DM^31^. A meta-analysis showed that the risk of developing colorectal cancer in type 2 diabetic patients is 20–40% greater than non-diabetic individuals^14^. However, conducting mass colorectal cancer screening using costly and/or invasive tests among all patients with type 2 diabetes mellitus would not be an effective approach because the vast majority of these patients do not have colorectal cancer-associated diabetes mellitus. Over-screening may lead to overtreatment and cause more harm than gain. In recent decades, the rapidly increasing incidence and relatively stable mortality of thyroid and prostate cancer in many countries, including China, the USA, and the UK could be partly due to overdiagnosis with ultrasonic or prostate-specific antigen screening tests^32^. By identifying high-risk patients for biomarker evaluation or conclusive diagnostic testing, our predictive model provides a low-risk and low-cost solution to this problem.

As far as we know, epidemiologic studies of colorectal cancer-associated type 2 diabetes mellitus have been limited to assessing individual predictors including age^29^, sex^33^, lifestyle factors^34^ (e.g. obesity, unfavorable diets, and low levels of physical activity) and blood-glucose-lowering drugs^35^. However, most findings from conducted studies may be possibly methodologically biased making it difficult to make an accurate prediction based on individual risk factors. Our findings indicate individualized patterns of demographic and clinical risk that can help identify patients with T2DM who may benefit from referral to services and over screening for colorectal cancer.

The most important and final contention for using the nomogram is the character need for further fitting or care. However, although the nomogram shows an overall excellent risk prediction performance, it still cannot accurately capture the clinical outcome of a certain level of discrimination or miscalibration^36^. Therefore, the decision curve analysis was performed to determine whether the nomogram judgment could be beneficial to explain the clinical utility. This method offers novel insight into clinical outcomes based on the threshold probability from which the net benefit can be derived^37^. The decision curve showed that applying the nomogram in our study to predict colorectal cancer provides more advantages than either the screen-all-patients or the screen-none scheme if the threshold prediction of a patient or doctor is 10–45%.

There are certain limitations to the nomogram presented herein. First of all, we constructed the prediction nomogram based on the retrospective review of medical records, the database did not included other risk factors for colorectal cancer such as dietary patterns and lifestyle changes such as exercising and consumption of processed meat. Thus, restricted in using certain factors such as regions and races may have limited the power of our nomogram to identify their significance. However, we can continuously adjust the parameters in actual application, making the nomogram better suited for practical application. Second, our nomogram should be viewed as a case-screeing approach to identify patients with T2DM who should receive additional definitive diagnostic testing but not as a diagnostic test. Third, given colorectal carcinoma normally develops from polyps taking 10–15 years, patients may already be living with pre-cancerous lesions at the time of T2DM diagnosis. However, because colonoscopy is an invasive examination, it is difficult to perform universally. Indeed, the final result is a simple and interpretable model for colorectal cancer prediction based on demographic and clinical variable, which , can be easily applied in the screening of patients with T2DM, with economical and low-risk. The forecasting model can be optimized based on additional databases including the severity of diabetes and wider geographic recruitment so as to achieve better prediction accuracy for future use.

## Conclusion

We have developed a nomogram that can predict the risk of colorectal cancer in patients with T2DM. The nomogram showed favorable calibration and discrimination values, which may help clinicians in making recommendations about colorectal cancer screening for patients with T2DM.
